# Parent–Child Relationships and Resilience Among Chinese Adolescents: The Mediating Role of Self-Esteem

**DOI:** 10.3389/fpsyg.2018.01030

**Published:** 2018-06-21

**Authors:** Lumei Tian, Lu Liu, Nan Shan

**Affiliations:** Department of Psychology, Shandong Normal University (SDNU), Jinan, China

**Keywords:** parental support, parent–adolescent conflict, resilience, self-esteem, adolescent

## Abstract

The present study primarily aimed to examine whether self-esteem serves as a mediator in the associations between parent–child relationships, including parental support and parent–child conflict, and resilience among adolescents. Three hundred and four Chinese adolescents were surveyed with questionnaires and structural equation modeling was adopted to test the mediational hypothesis. The results indicated that the associations between parent–child relationships and adolescent resilience were primarily mediated by self-esteem and that parental support was more robustly linked with adolescent resilience than parent–adolescent conflict. The current study also tested a competitive mediational model in which resilience was the mediator and self-esteem was the outcome variable, and observed that this model was also well-established but inferior to the hypothesized mediational model. These findings extend our insight into the mechanisms underlying the associations among parent–child relationships, self-esteem, and resilience among adolescents and suggest that adolescent resilience promotion programs should focus on improving parental support in a family context and developing individual self-esteem.

## Introduction

Adolescence is characterized by rapid physical changes along with social and psychological challenges. For instance, there is a dramatic increase in the prevalence of emotional symptoms, such as anxiety and depression (Skrove et al., 2013). Adolescents also suffer from high stress and challenges from academic performance. Resilience, however, as a foundation for positive development in adolescence (Wright and Masten, 2005), is likely to facilitate young people’s mental health (Hu et al., 2015) and well-being (Liu et al., 2012). Thus, it is essential to explore the factors that may predict adolescent resilience and the mechanism underlying their relationships, which would help to develop interventions aimed at improving adolescents’ positive development. According to the ecological systems theory (Bronfenbrenner, 1979) and the resilient systems model (Mandleco and Peery, 2000), both environmental factors (e.g., family) and individual characteristics play vital roles in adolescent development. Consequently, the present study examined the associations of parent–child relationships (as an important environmental factor) and self-esteem (as a critical individual characteristic) with adolescent resilience and the mechanism underlying them.

Various definitions of resilience have been proposed (Kaplan, 1999). Resilience is usually considered from three perspectives: outcome, process, and personality. For example: (a) the positive developmental outcomes among individuals at high risk (Rutter, 1990); (b) a dynamic process of positive adaptation when exposed to a significant threat or severe adversity (Luthar et al., 2000); (c) a set of traits reflecting general resourcefulness and sturdiness of character and flexibility of functioning in response to various environmental circumstances (Block and Block, 1980). Although there has been less consensus relating to these definitions, resilience is expressed in continuing to live strongly in spite of hardships. For intervention purposes, resilience was defined as a coping process in the present study and would be measured using a questionnaire corresponding to this definition, since neither personalities nor outcomes can be easily altered. In recent years, interest in investigating this concept among children and adolescents has increased because of the increasing influences from positive psychology. Resilience has been believed to be a foundation for positive development in childhood and adolescence (Wright and Masten, 2005) and to be essential to facilitating young people’s mental health (Hu et al., 2015) and well-being (Liu et al., 2012). Resilience has thus become a focus of previous research of adolescent development.

There are three interrelated domains pertaining to the individual, the familial and the social environment promoting individual resilience (Newman, 2002; Olsson et al., 2003; Schofield and Beek, 2005; Sippel et al., 2015; see review, Gilligan, 2004; Stein, 2008). These domains were influenced by internal factors (biological and psychological factors) and external factors, suggested by the resilient system model (Mandleco and Peery, 2000). Similarly, it has been believed that an individual’s resilience derives not only from innate characteristics but also from external circumstances (Cicchetti and Valentino, 2006).

Among external factors, family has the most direct and lasting effect on the normal development of adolescents according to Bronfenbrenner’s (1979) ecological systems theory and Bowlby’s (1971) attachment theory. Yates and Masten (2004) also refined the developmental model of resilience to focus on factors that shape developmental pathways, including influence from family. Research has found that contextual positive factors (e.g., service use) or risks predict adolescent resilience (Sanders et al., 2015). Regarding the family, parental support, the positive aspect of the parent–adolescent relationship, is considered an essential feature in the normal development of adolescents. This support is generally conceptualized as a care resource that can be received from parents, such as parents’ emotional support (Huver et al., 2010), instrumental support or assistance in handling a problem (Pierce et al., 1996). A positive parent–child relationship has been found to positively predict adolescent resilience (Werner, 2000; Brennan et al., 2003). It should be noted that family relationships are often characterized by high levels of conflict for both males and females, particularly during adolescence (Furman and Buhrmester, 1992). Even conflict between parents and adolescents is very common during adolescence (Laursen et al., 1998). Although parent–adolescent conflict is adaptive and reflects adolescents’ desire for independence from parents (Smetana, 1989; Sorkhabi, 2010), it results in an ambivalent relationship between adolescents’ and parents’ discrepant expectations regarding appropriate behavior and the timing of transitions in authority, autonomy, and responsibilities (Dekovic et al., 1997); therefore, the negative effect of parent–child conflict is higher during adolescence than during other age periods (Laursen and Collins, 2009). Parent–child conflict is usually regarded as a negative aspect of the relationship (Smokowski et al., 2015). As advanced by McLoyd (1990) in the Family Stress Model, a poor parent–child relationship is a family stressor on subsequent child outcomes. A high level of parent–adolescent conflict has been found to be associated with adolescents’ mental health symptoms (Repetti et al., 2002) and problem behavior (Yeh, 2011). In summary, parental support has been believed to have a positive effect on the development of the individual’s internal resources (Kumpfer and Summerhays, 2006) and to be a good support system to promote the development of adolescent resilience (Ozbay et al., 2008; Wu et al., 2012; Dawson and Pooley, 2013), while the conflict in the family has been thought to be a risk factor for resilience in children and youth (Zolkoski and Bullock, 2012).

Among internal factors, self-esteem should be another important source of resilience. Although there are many definitions and different types of self-esteem in psychology, unless stated otherwise, self-esteem is usually defined as a set of one’s own thoughts and feelings about his or her worth and importance (Rosenberg, 1965) or the general evaluation and appraisal of one’s worth (e.g., Leary and Baumeister, 2000; Hewitt, 2009). From the perspective of Terror Management Theory (TMT, Greenberg et al., 1986), self-esteem serves an anxiety-buffering function, which has been supported empirically by experimental studies both within the Western (Greenberg et al., 1992; Schmeichel et al., 2009) and Chinese context (Zhang and Tian, 2005; Zhang et al., 2005). In this sense, self-esteem protects individuals from anxiety and thereby contributes to their positive development in terms of cognition and emotion (Ford and Collins, 2010), subjective well-being (Orth et al., 2012), and mental health and social behavior (Hosman et al., 2004). Several studies (Martinez-Torteya et al., 2009; Benzies and Mychasiuk, 2010) indicated that resilience could be promoted by protective factors and inhibited by risk factors. Self-esteem is such an individual internal protective factor (Haase, 2004; Veselska et al., 2009; Pan and Yang, 2013) and psychological resources that can be used to explain the overall structure of resilience (Windle et al., 2008). Self-esteem has also been found to have a certain predictive effect on resilience (Glenna and John, 2012; Bajaj, 2017; Balgiu, 2017; Martínez-Martí and Ruch, 2017). Self-esteem is a critical internal source of resilience of adolescents. However, the relationship between self-esteem and resilience is more complex (Miller and Daniel, 2007). In other words, resilience also exerts an impact on self-esteem (Benetti and Kambouropoulos, 2006; Mak et al., 2011; Liu et al., 2014). Theoretically, resilience, as a foundation for positive development in childhood and adolescence (Wright and Masten, 2005), can be inferred to affect adolescent self-esteem, which is included in mental health indicators (Newland, 2014). Resilience has been found to facilitate adolescent self-esteem (Benetti and Kambouropoulos, 2006; Mak et al., 2011; Liu et al., 2014). Accordingly, there may be a bidirectional relationship between self-esteem and resilience. Not only does self-esteem affect the resilience, but resilience may influence self-esteem, as well.

Additionally, parents or parenting exert an extensive and far-reaching influence on adolescent development. The association between parent–child relationships and self-esteem is well-documented. For example, Bowlby’s (1971) attachment theory states that a child will be presumed to form a stable internal working model of self and others, which will cognitively represent the early pattern of parental responsiveness to his or her bids for care and support. A child develops a feeling of lovableness or unlovableness in his or her model of the self, which in turn becomes a part of his or her self-esteem. In other words, through consistent, warm, and supportive interactions with a caregiver, a child is thought to develop an internal working model that consists of positive views of the self (Thompson, 2006). Although adolescents spend less time with their parents and their relationships with parents are going through a period of reorganization (e.g., independence), there is no reason to believe that parental support is unimportant for adolescent self-esteem (Harter, 2006). Indeed, a close and supportive parent–child relationship is still an important source of adolescents’ self-esteem (Mattanah et al., 2011). In contrast, parent–child conflict is usually regarded as a negative aspect of the parent–child relationship (Smokowski et al., 2015) and is expected to serve as a risk factor against self-esteem (Cotter et al., 2013).

Such views have been supported by a large body of empirical research. Parental support has been found to be positively linked to adolescents’ self-esteem in both Western (Rueger et al., 2010; Behnke et al., 2011; Mcmahon et al., 2011; Boudreault-Bouchard et al., 2013; Kerpelman et al., 2016) and Chinese society (Tian et al., 2013; Wang et al., 2016). Maternal and paternal emotional support even reinforces adolescents’ self-esteem over time (Boudreault-Bouchard et al., 2013). These findings therefore underscore the idea that parental support is among the strongest predictors of adolescent self-esteem. A great deal of research has also shown an inextricable negative relationship between parent–adolescent conflict and self-esteem, including longitudinal studies (e.g., Smokowski et al., 2010; Juang et al., 2012) and cross-sectional studies (Caughlin and Malis, 2004; Sun et al., 2006; Kuhlberg et al., 2010; Ozdemir, 2014). Thus, it can be seen that parental support is a protective factor whereas parent–adolescent conflict is a risk factor for adolescent self-esteem development.

**FIGURE 1:**
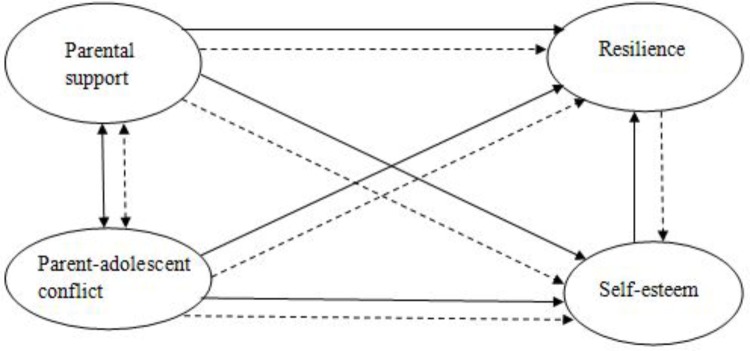
Hypothesized/competitive model. The solid line indicates the hypothesized mediational model with self-esteem as a mediator. The dotted line indicates the competitive mediational model with resilience as a mediator.

Taken together, parental support and parent–adolescent conflict are two major family context factors associated with adolescent resilience and self-esteem. Self-esteem is an individual psychological resource of adolescent resilience, as well. We assumed that self-esteem would play a mediating role in the associations between parent–child relationship and resilience of adolescents (H1). In other words, a good parent–child relationship would foster healthy self-esteem which, in turn, would promote resilience. However, the two aspects of a parent–child relationship, support and conflict, might be differentially related to adolescent development, while few studies have taken them into account simultaneously, especially with Chinese samples. Against this background, we sought to fill this gap by examining them within a same mediational model in the current study. Given that a close and supportive parent–child relationship is an important source of adolescent self-esteem (Mattanah et al., 2011) and positive family experiences play a critical role in shaping self-esteem in children and adolescents (Shaffer and Kipp, 2010) while parent–adolescent conflict is primarily a risk factor against adolescent development, we hypothesized that parental support would be more strongly linked to adolescent resilience, compared with parent–adolescent conflict (H2). Lastly, there should be a reciprocal relationship between self-esteem and resilience as mentioned above. Thus, we also established a competitive model in which resilience was the mediator in the association between the parent–child relationship and adolescent self-esteem and hypothesized that this mediational model would also be valid but the mediational model with self-esteem as the mediator would be superior to it, based on the aforementioned theoretical frameworks and empirical findings (H3). The hypothesized mediational model and the competitive one are depicted in **Figure 1**.

## Materials and Methods

### Participants

The participants were recruited from a public high school located in the center of Jinan, Shandong Province, China. A total of 312 Chinese adolescents were invited as the initial sample. Among these adolescents, 304 (149 females and 155 males) completed the questionnaires, giving a response rate of 97.44%. Eight cases were excluded due to missing data. The average age of the participants was 16.93 years (*SD* = 0.84). All of the participants were Han Chinese, which made up 91.60% of the total population in the 2015 population census of China from the National Bureau of Statistics of People’s Republic of China. The sample covered 113 (37.17%) students in the 10th grade, 92 (30.26%) in the 11th grade, and 99 (32.57%) in the 12th grade. Moreover, there were 257 (84.54%) only children and 47 (15.46%) participants with at least one sibling in the family. A total of 285 adolescents (93.75%) lived with both parents, and 19 adolescents (6.25%) lived with a single parent.

### Measures

#### Parent–Child Relationship: Parental Support and Parent–Adolescent Conflict

Perceived parental support and parent–adolescent conflict were assessed with the Network of Relationships Inventory (NRI; Furman and Buhrmester, 1985), which measures the perceptions of the quality of the relationship with the father and mother. Participants answered all questions about their relationships with a mother figure (i.e., natural mother, stepmother, adoptive mother or other important mother figure) and a father figure (i.e., natural father, stepfather, adoptive father or other important father figure). The Chinese shortened version of the NRI was adapted by Tian et al. (2012). Five dimensions, each with three items, were assessed: companionship (e.g., “How much do you and this person spend free time together?”), instrumental help (e.g., “How much does this person help you figure out how to fix things?”), intimacy (e.g., “How much do you talk to this person about things you do not want others to know?”), affection (e.g., “How much does this person like or love you?”), and conflict (e.g., “How much do you and this person disagree and quarrel?”). Each item was administered successively for both relationship types and was rated on a five-point Likert scale (1 = “Little or None,” 5 = “the Most”). The former four dimensions from both parents were combined into a composite averaged parental support score, according to the procedure in previous studies (Rubin et al., 2004; Tian et al., 2012). Higher scores represent more perceived parental support. Scores on the conflict dimension from both parents were also combined into a composite averaged parent–adolescent conflict score, with higher scores representing higher levels of parent–adolescent conflict. In the present study, the Cronbach’s alpha coefficient was 0.88 for the whole NRI, 0.92 for the parental support and 0.88 for the parent–adolescent conflict.

#### Self-Esteem

Self-esteem was assessed with the 10-item Rosenberg Self-Esteem Scale (RSES; Rosenberg, 1965), which comprises five positively and five negatively worded items. Each item was rated on a four-point scale ranging from 1 (strongly disagree) to 4 (strongly agree), with higher mean scores indicating higher levels of self-esteem. The Chinese version of the RSES was translated by Ji and Yu (Wang et al., 1999), and good validity and reliability among Chinese adolescents have been confirmed (Li et al., 2010; Mak et al., 2011), except that there was cultural difference in understanding the eighth item in this questionnaire and thus it was not appropriate for Chinese adolescents (Tian, 2006). Accordingly, the eighth item was excluded in later analyses. The Cronbach’s alpha coefficient in the present study was 0.87.

#### Resilience

The Adolescent Resilience Scale (ARS; Hu and Gan, 2008), a localized resilience scale with good validity and reliability, was adopted in the present study. This scale was designed to emphasize the measurement of the adolescent coping process with adversity involving cognition, emotion, behavior, and a support system. The scale consists of 27 items that fall into five subscales, respectively, representing goal planning (five items, e.g., “After setbacks, I usually become more mature and experienced.”), help-seeking (six items, e.g., “I do not know whom to ask for help when I am in trouble.”), family support (six items, e.g., “No one would like to listen to what I say in my family.”), affect control (six items, e.g., “I can adjust my mood well in a short time.”), and positive thinking (four items, e.g., “I believe that adversities can stimulate or motivate people to make progress.”). For each statement, participants rated items on a five-point Likert scale, from 1 (completely disagree) to 5 (completely agree). A higher averaged score indicated that resilience was greater. Because the family support dimension confounds with parental support from NRI to a certain degree in the current study, we excluded this dimension in the following analyses. The Cronbach’s alpha coefficients were 0.72 for the remaining items and 0.60–0.68 for the four subscales in the current study.

### Procedure

The data in the current study were collected by trained graduate students in school classrooms without teachers being present after the school granted permission for the study to be performed. Prior to survey administration, the students were informed that participation was voluntary and that they could either refuse to participate in or withdraw from the study at any time. Although active consent procedures (e.g., written informed consent) satisfy legal and ethical requirements, because they, particularly active parental consent procedures, include such problems as low response rates, non-representative samples, and costly implementation (Hollmann and Mcnamara, 1999) and even biased estimates of associations between outcomes of interest (Shawa et al., 2015), the passive consent procedures recommended by Ellickson (1989) were adopted in the present study. Passive consent procedures ask students and their parents to return a form only if they do not want (their children) to participate in the research. Those who do not return the form are assumed to consent to (let their children) participate in the research. All participants were given the three structured, anonymous, and self-reported questionnaires in a classroom setting. To ensure a correct understanding of the questions and responses, the trained graduate students walked around in the classroom to help those who had any difficulties understanding the questions or responses. Participants did not write their names on the questionnaires and were assured of data confidentiality. All measures were completed in approximately 25 min. The study protocol was approved by the Institutional Review Board at Shandong Normal University in China.

### Data Analyses

The data were mainly analyzed using structural equation modeling (SEM) in AMOS 20.0 software. SEM procedures were used to examine the mediating role of self-esteem/resilience in the relationship between parent–child relationship and resilience/self-esteem among adolescents. The advantage of this procedure is the reduction of type I error, compared with a series of regression analyses in which all equations are conducted simultaneously. The analysis proceeded in several steps. First, to determine the relationship between the indicators and the latent constructs (i.e., measurement models), a confirmatory factor analysis (CFA) was assessed. Second, the structural models were tested with SEM to examine the relationship between the independent (parent–adolescent relationship) and the dependent variable (i.e., model 1) and the mediating roles of self-esteem/resilience when the mediator was entered into the model (i.e., model 2).

In addition, several authors have argued that item parceling is a beneficial practice within the SEM framework (Little et al., 2002). More specifically, combining items into small groups of items within a scale or subscale has been demonstrated to increase the stability of the parameter estimates and improve the variable to sample size ratio. The latent variable for self-esteem was created using the item-to-construct balance technique (Little et al., 2002), according to previous research (Tsigilis and Srebauite, 2015), because the self-esteem scale has only a single dimension. Three item parcels were thus created for self-esteem. The indicators of parent–adolescent conflict were indicated by the three items of the conflict subscale, and parental support was comprised of four dimensions as its four indicators: (a) companionship, (b) instrumental help, (c) intimacy, and (d) affection. Resilience was comprised of the four indicators (i.e., the four subscales of the ARS).

Next, using the maximum-likelihood program, we estimated path coefficients and estimated the fit of each model to the data. The following goodness-of-fit indices were used: chi-square statistics; *χ^2^/df* ratio, comparative fit index (CFI), goodness-of-fit index (GFI), Tucker–Lewis index (TLI), and the Mean Square Error of Approximation (RMSEA). The model is considered to have an acceptable fit if *χ^2^/df* < 3. For CFI, GFI and TLI, values greater than 0.90 indicate an acceptable model fit. For RMSEA, a value between 0.08 and 0.10 shows a mediocre fit and a value below 0.08 indicates a good fit (MacCallum et al., 1996). Additionally, Akaike Information Criterion (AIC) and Bayesian Information Criterions (BIC) were used to compare the two mediational models. The smaller the values of AIC and BIC are, the better the model. Lastly, if the mediational model was valid, to further corroborate whether the chosen variable was a mediator, maximum likelihood bootstrapping was used wherein confidence intervals (95%) for the indirect effect were estimated (1,000 samples were drawn). Statistical significance was determined with 95% bias-corrected bootstrapped confidence intervals that did not contain zero.

## Results

The common-method bias might occur because all data in the present study came from adolescents’ self-reports. Thus, prior to further data analyses, a CFA of a single-factor model comprised of 14 observed variables was conducted and provided a poor fit to the data (*χ*^2^ = 933.25, *df* = 77, *p* < 0.001, *χ*^2^/*df* = 12.12, RMSEA = 0.19, CFI = 0.50, GFI = 0.68, TLI = 0.41). Consequently, there was no serious common-method bias in the current study.

### Descriptive Statistics

Means, standard deviations, and Pearson’s correlations among principal study variables are presented in **Table 1**. As shown, all variables were significantly related to each other (*p*s < 0.01). Either self-esteem or resilience was positively correlated with parental support, but negatively associated with parent–adolescent conflict. Moreover, significant positive correlations were observed between resilience and self-esteem as well. These findings provided preliminary evidence for the hypothesized relations among variables, and allowed for further analyses to examine the hypothesized mediational model. We also tested the gender differences, grade differences, sibling status differences, and family status differences using *t*-tests, non-parametric *U*-tests, or one-way ANOVAs, and no significant differences in self-esteem and resilience were found (*p*s > 0.05). Thus, these demographic variables were not considered in further analyses.

**Table 1:** Correlations, means, and standard deviations for the variables (*N* = 304).

Variables	Mean (*SD*)	1	2	3
1. Parental support	7.08 (1.32)	—		
2. Parent–adolescent conflict	4.43 (1.66)	0.19^∗∗^	—	
3. Resilience	3.38 (0.49)	0.19^∗∗∗^	-0.17^∗∗∗^	—
4. Self-esteem	3.14 (0.51)	0.31^∗∗∗^	-0.30^∗∗∗^	0.46^∗∗∗^

*^∗∗^p < 0.01, ^∗∗∗^p < 0.001.*

### Structural Equation Model Analyses

First, the measurement model was established, comprised of four latent factors (parental support, parent–child conflict, self-esteem, and resilience) and 14 observed variables. The preliminary CFA of the measurement model provided a good fit to the data. *χ*^2^ = 179.01, *df* = 71, *p* < 0.001, *χ*^2^/*df* = 2.52, RMSEA = 0.07, CFI = 0.94, GFI = 0.92, TLI = 0.92. Significant factor loadings were found for the indicators on the latent variables, indicating that the latent factors were well represented by their respective indicators.

**FIGURE 2:**
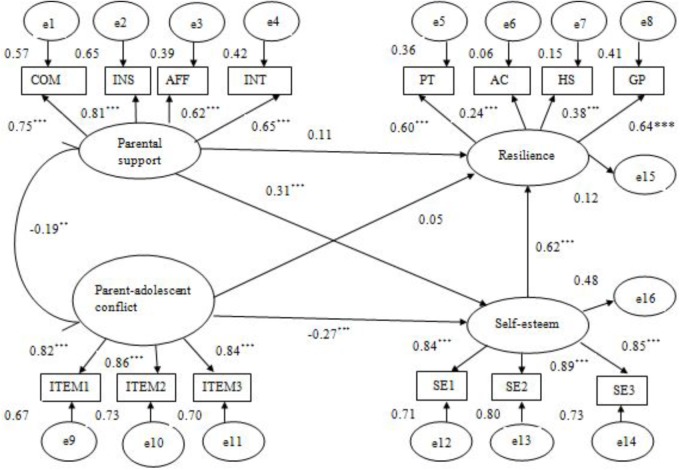
Standardized path coefficients in the final mediational model with self-esteem as a mediator (*N* = 304). COM, companionship; INS, instrumental help; AFF, affection; INT, intimacy. ITEM1–ITEM3, three items measuring parent–adolescent conflict from NRI. PT, positive thinking; AC, affect control; HS, help seeking; GP, goal planning. SE1–SE3, three item parcels from the Self-Esteem Scale. ^∗^*p* < 0.05, ^∗∗^*p* < 0.01, ^∗∗∗^*p* < 0.001.

Next, to test H1 and H2, model 1 was established, in which resilience was the outcome variable and both parental support and parent–adolescent conflict were the predictor variables. Then, the mediating variable (self-esteem) was entered into the model to build model 2 (**Figure 2**). The fit indices indicated that model 1 with all direct paths between study variables showed a mediocre fit to the data: *χ*^2^ = 140.97, *df* = 41, *p* < 0.001, *χ*^2^/*df* = 3.44, RMSEA = 0.09, CFI = 0.90, GFI = 0.92, TLI = 0.87, whereas the hypothesized model 2 had a good fit to the data:*χ*^2^ = 179.01, *df* = 71, *p* < 0.001; *χ*^2^/*df* = 2.52, RMSEA = 0.07, CFI = 0.94, GFI = 0.92, TLI = 0.92. Furthermore, in model 1, the standardized path coefficient from parental support to resilience was statistically significant (β = 0.28, *p* < 0.01), but the path from parent–adolescent conflict to resilience was not (β = -0.11, *p* > 0.05), which explained 48.28% and 18.97% of the resilience variance, respectively. In model 2, the standardized path coefficient either from parental support or from parent–adolescent conflict to resilience was not statistically significant (βs = 0.11, 0.05, *p*s > 0.05). Interestingly, both the indirect effect of parental support and the effect of parent–child conflict via self-esteem were statistically significant (β = 0.19, *p* < 0.01, 95% CI [0.11, 0.31]; β = -0.17, *p* < 0.01, 95% CI [-0.28, -0.07]) and greater than their direct effects (βs = 0.11, 0.05) on resilience among adolescents, the former explaining approximately 63.33% of total effects (0.30) and the latter even bigger than its total effect (-0.12). These findings indicated that the association between parent–child relationships, either support or conflict, and adolescent resilience was primarily mediated by self-esteem and that parental support was more strongly linked with resilience than parent–adolescent conflict.

**FIGURE 3:**
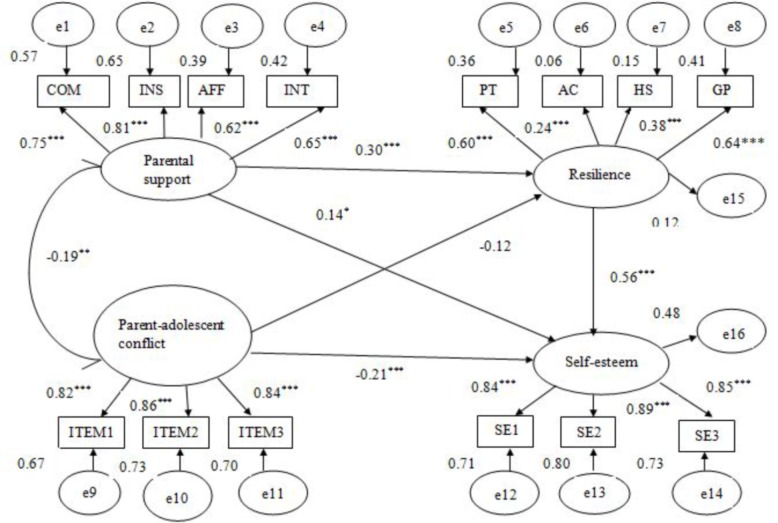
Standardized path coefficients in the final mediational model with resilience as a mediator (*N* = 304). COM, companionship; INS, instrumental help; AFF, affection; INT, intimacy. ITEM1–ITEM3, three items measuring parent–adolescent conflict from NRI. PT, positive thinking; AC, affect control; HS, help seeking; GP, goal planning. SE1–SE3, three item parcels from the Self-Esteem Scale. ^∗^*p* < 0.05, ^∗∗^*p* < 0.01, ^∗∗∗^*p* < 0.001.

To test H3, we ran another SEM in which resilience was the mediating variable and self-esteem was the outcome variable. Similarly, model 1 was first established, in which self-esteem was the outcome variable and both parental support and parent–adolescent conflict were the predictor variables. Next, the mediating variable (resilience) was entered into the model to build model 2 (**Figure 3**). The analyses indicated that model 1 with all direct paths between study variables had a mediocre fit to the data: *χ*^2^ = 128.21, *df* = 32, *p* < 0.001, *χ*^2^/*df* = 4.01, RMSEA = 0.10, CFI = 0.94, GFI = 0.92, TLI = 0.91, whereas model 2 had a good fit to the data: *χ*^2^ = 179.01, *df* = 71, *p* < 0.001, *χ*^2^/*df* = 2.52, RMSEA = 0.07, CFI = 0.94, GFI = 0.92, TLI = 0.92. Further, in model 1, the standardized path coefficients both from parental support (β = 0.31) and from parent–adolescent conflict to self-esteem (β = –0.27) were statistically significant (*p*s < 0.001), which explained 39.74% and 34.62% of the self-esteem variance, respectively. In model 2, however, the standardized path coefficient from parental support to self-esteem was still significant but decreased (β = 0.14, *p* < 0.05), while the path coefficient from parent–adolescent conflict to self-esteem was also significant and only slightly smaller (β = –0.21, *p* < 0.001). Importantly, the indirect effect of parental support through resilience was statistically significant (β = 0.17, *p* < 0.01, 95% CI [0.04, 0.38]) but not noticeably different from its direct effect (β = 0.14) on self-esteem, explaining approximately 54.84% of total effects, whereas the indirect effect of parent–adolescent conflict (-0.07) via resilience was not statistically significant (β = -0.07, *p >* 0.05, 95% CI [-0.19, 0.03]) and was smaller than its direct effect (β = -0.21) on self-esteem, explaining only 25% of total effects. These findings indicated that resilience partially mediated the relationship between parental support and self-esteem but did not mediate the association of parent–adolescent conflict with self-esteem.

In summary, the two mediational models were both well-established, but the hypothesized model with self-esteem as the mediator (AIC = 228.57, BIC = 328.93) was superior to the competitive model with resilience as the mediator (AIC = 247.01, BIC = 373.39)^1^. The mediating roles of self-esteem in the hypothesized model were notably significant and dominating, whereas the mediating roles of resilience in the competitive model were highly limited.

## Discussion

The main goal of this present study was to examine the associations of parent–child relationships, including parental support and parent–child conflict, with adolescent resilience and the mediating role of self-esteem. Overall, the results indicated that the integrated mediational model was well-established, consistent with our H1. Specifically, in this model, the indirect effects of both parental support and parent–child conflict on adolescent resilience were statistically significant and greater than their direct effects, neither of which was statistically significant. These findings showed that the associations of parent–child relationships and the resilience of adolescents were primarily mediated by individual self-esteem. Interestingly, parental support was significantly related to adolescent resilience whereas the relationship between parent–child conflict and resilience was not obvious, in accordance with our H2.

In line with some developmental theories, such as Bowlby’s (1971) attachment theory, Mandleco and Peery’s (2000) resilient system model, McLoyd’s (1990) Family Stress Model and previous research (Brennan et al., 2003; Ozbay et al., 2008; Dawson and Pooley, 2013), parent–child relationships, particularly parental support, provide a solid foundation for individual development. As a protective factor, parental support has a positive effect on the development of the individual’s internal resources (Kumpfer and Summerhays, 2006) and is a good support system to promote the development of adolescent resilience. Even though parental influences might weaken because teenagers have an increasing need of independence and spend less time with parents during adolescence, support from parents is still an important external source of adolescent resilience. Additionally, consistent with our expectation and previous findings (Rueger et al., 2010; Tian et al., 2013; Wang et al., 2016), we observed that parental support was significantly and positively related to the self-esteem of adolescents. Importantly, self-esteem mediated the majority of the associations between parental support and resilience. Adolescents are forming views about themselves and experiencing others’ evaluations at the same time, which could affect their self-esteem development. Support from parents is a critical source of youth self-esteem in the long run (Harter, 2006). This finding is also in line with the attachment theory advanced by Bowlby (1971) which states that parents’ trust, support and tolerance for their children will help them develop a positive internal working model. A good support system from parents helps adolescents form high self-esteem. On the other hand, resilience is derived from both external and internal factors (Cicchetti and Valentino, 2006). Self-esteem is a very crucial internal protective factor of resilience (Haase, 2004; Pan and Yang, 2013) and is a psychological resource that can be used to explain the overall structure of resilience (Windle et al., 2008). This property of self-esteem can be interpreted within the framework of TMT proposed by Greenberg et al. (1986), which states that self-esteem serves as an anxiety buffer. Self-esteem protects individuals from anxiety and thereby contributes to positive adolescent development, including facilitating resilience. In summary, self-esteem can robustly mediate the links between parent–child relationships and the resilience of adolescents.

Additionally, in accordance with our H2, we observed that parental support was more robustly related to adolescent resilience than parent–child conflict. This finding was noteworthy, as it indicated that interventions to promote adolescent resilience need to place greater stress on parental support in the future. This result was observed presumably because parent–adolescent conflict is not necessarily negative in nature in the sense that it also means adolescents’ gradually regarding themselves as independent and autonomic and primarily concerning peer group conventions, rather than parental ones, as a normal developmental process (Smetana et al., 2006). However, conflict is often related to an adolescent’s poor adjustment (Yeh et al., 2010). The dual roles of parent–adolescent conflict may mitigate its relationships with some developmental outcomes. The current study found that the direct effect of parent–adolescent conflict on resilience was positive while its indirect effect and total effect were both negative. On the contrary, a supportive parent–child relationship is an important source of adolescent self-esteem (Mattanah et al., 2011) and plays a critical role in shaping self-esteem in children and adolescents (Shaffer and Kipp, 2010).

Finally, consistent with our H3 and prior studies (Benetti and Kambouropoulos, 2006; Miller and Daniel, 2007; Mak et al., 2011; Liu et al., 2014), resilience and self-esteem have a reciprocal relationship. The competitive mediational model with resilience as a mediator was also well-established in the current study. The resilience, however, only partially mediated the association of parental support, but not parent–adolescent conflict, with self-esteem of adolescents. Further, this indirect effect of resilience was not noticeably different in magnitude or direction from its direct effect. These findings indicated that the mediational model with self-esteem as a mediator was superior to the model with resilience as a mediator in which the parent–child relationship was primarily linked directly to adolescent self-esteem. Theoretically, resilience, as a foundation for positive development in childhood and adolescence (Wright and Masten, 2005), can be inferred to affect adolescent self-esteem, which is included in mental health indicators (Newland, 2014). Accordingly, they may be reciprocal and influence each other mutually. Even so, as an internal source, self-esteem is more likely to have a great effect on resilience rather than the opposite. This conclusion, however, should be accepted with caution before it is further examined, and more supportive evidence, particularly experimental or quasi experimental evidence, should be sought in future studies.

Taken together, the data support our hypotheses and provide evidence that the parent–child relationship, particularly parental support, plays a vital role in adolescent resilience and self-esteem primarily mediates their relationship, suggesting that parental support and self-esteem are crucial sources of adolescent resilience.

### Limitations and Implications

This study has several limitations. First, all data in this study came from the adolescents’ self-reports. Even though a self-report can more accurately reflect the level of a perceived construct (e.g., self-esteem) than other reports, the single-subject assessment may be less abundant, since self-report measures are easily influenced by social desirability, which might discount the reliability of data. Future research should utilize multiple assessment methods (e.g., peer report) to improve the quality of the data and thereby the validity of the findings. Second, the current study adopted a cross-sectional design, which makes it difficult to draw any causal relationship among the variables. Therefore, longitudinal designs or experimental/quasi experimental designs are needed to provide more reliable conclusions about the directionality of these effects (e.g., do positive relationships with parents increase resilience or self-esteem?) in the future. Third, while the overall results were statistically significant, the sample in the present study was not representative enough, due to its small size and limited sampling scope, which might limit the generalizability of the findings. Future studies should expand the sample size and sampling scope (e.g., using a national sample) to further test the generalizability of the findings. Lastly, this study did not collect data about the participants’ (or their parents’) socioeconomic status, which might be related to adolescents’ development outcomes (e.g., the higher socioeconomic status is, the higher self-esteem may be), though we did not think it necessarily influence the associations among the outcomes (e.g., it does not necessarily moderate the association between self-esteem and resilience). This demographic factor should be considered in future studies.

Despite these caveats, the findings of the current study provide valuable information. First, this study simultaneously examined the mediating roles of self-esteem in the relationships between both parental support and parent–adolescent conflict and adolescent resilience within the same model. The findings of the present study extend our insight into the mechanisms underlying the associations among parent–child relationships, self-esteem, and the resilience of adolescents and supplement previous relevant studies. Second, the current study found that self-esteem could positively predict adolescent resilience and vice versa, which provides further support for the reciprocal association between self-esteem and resilience. Finally, the findings of the present study suggest that adolescent resilience promotion programs should focus on adolescent parental support and self-esteem, particularly the improvement of parental support in a family context. After a comprehensive literature review, our study is among the first to indicate the mediating role of self-esteem in the relationship between parent–child relationship, both support and conflict, and resilience. It is also important for adolescents to boost self-esteem training, cultivation, development, and promotion.

## Author Contributions

LT provided much support and guidance in the research and revised the manuscript substantially. LL and NS conducted measurements, data collection and data analysis, and revised the manuscript. All authors approved the final version.

## Conflict of Interest Statement

The authors declare that the research was conducted in the absence of any commercial or financial relationships that could be construed as a potential conflict of interest.

## Footnotes

1 We also tested other possible mediational models in which both parental support and parent–child conflict were either dependent variables or mediators, and found them to be similarly acceptable statistically and no obvious inferiority (AIC = 246.22–248.57, BIC = 249.66–369.11) to the hypothesized mediational model was detected, suggesting that relationships among the variables of interest are intricate and possibly influence each other. Theoretically, however, the parent–child relationship is usually thought to be the basis and the source of children’s development according to Bowlby’s (1971) attachment theory. Therefore, we did not present these detailed results and will not discuss them below.
